# A narrative review of music therapy for neuropsychiatric symptoms in Alzheimer’s disease and rationale for protocolized music teletherapy

**DOI:** 10.3389/fmed.2023.1248245

**Published:** 2023-11-21

**Authors:** Sonya G. Wang, Andrea M. Cevasco-Trotter, Michael J. Silverman, Shauna H. Yuan

**Affiliations:** ^1^Department of Neurology, School of Medicine, University of Minnesota, Minneapolis, MN, United States; ^2^School of Music, The University of Alabama, Tuscaloosa, AL, United States; ^3^School of Music, University of Minnesota, Minneapolis, MN, United States; ^4^Geriatric Research Education and Clinical Center (GRECC), Veterans Affairs Minneapolis Healthcare System, Minneapolis, MN, United States

**Keywords:** Alzheimer’s, dementia, geriatrics, music therapy, narrative review, telehealth, neuropsychiatric symptoms

## Abstract

**Introduction:**

Alzheimer’s Disease (AD) constitutes a major societal problem with devastating neuropsychiatric involvement in over 90% of those diagnosed. The large spectrum of AD neuropsychiatric symptoms leads to polypharmacological prescribing that, in turn, poses a major risk for increased side effects. Non-pharmacological interventions such as music therapy (MT) are therefore recommended as first-line treatments. The amalgamation of an aging population, long lifespan, and shortage of qualified music therapists limits access to MT services for AD.

**Objective:**

The purpose of this paper is to provide a rationale for a protocolized music teletherapy (MTT) intervention to increase accessibility for MT as a psychosocial intervention for neuropsychiatric symptoms in people with AD by conducting a narrative review of the existing MT and AD literature.

**Methods:**

We conducted a narrative review of MT and MTT publications indexed in PubMed and Google Scholar wherein authors used the Neuropsychiatric Inventory. We examined the impact of MT on neuropsychiatric symptoms of AD and identified MTT as a way to increase access to clinical services.

**Results:**

MT can have positive impacts on neuropsychiatric symptoms in AD. However, we identified an ensuing need for protocolized MT interventions, access to services, and increased awareness. MTT is an option that can address these needs.

**Discussion:**

Although MT can have positive effects on neuropsychiatric symptoms and can be beneficial and safe for individuals with AD, the current approach to MT practice is enormously heterogeneous with studies demonstrating variable therapist qualifications, uses of music, therapy approaches, and clinical populations. Congruently, the existing literature indicates that MT has not been standardized with protocolized interventions, making it difficult for clinicians and researchers to objectively assess the evidence, and thus, prescribe MT interventions. The lack of MT standardization, coupled with a low number of music therapists relative to people with AD, result in a lack of awareness that hinders access to MT as a psychosocial treatment for neuropsychiatric symptoms in people with AD. We therefore propose that protocolized MTT interventions are needed to increase access to better address neuropsychiatric symptoms associated with AD.

## Introduction

The Alzheimer’s Disease (AD) population in the United States is currently at 6.5 million and is predicted to reach over 12.5 million by 2050 (1). As such, AD constitutes a major societal problem warranting treatment, funding, and research. AD is accompanied by neuropsychiatric symptoms in over 85% of those diagnosed (2–5). These symptoms can be disruptive, impair care, and result in unsafe conditions for people with AD (6). Neuropsychiatric changes including irritability, agitation, anxiety, and depression are common in patients in the earlier stages of AD (2). In later AD stages, symptoms can include aggression, anger, restlessness, delusions, hallucinations, sleep disturbances, and sundowning. Additionally, environmental factors including but not limited to noise, heat, and social interactions, can activate and augment these symptoms (7, 8). These neuropsychiatric changes decrease quality of life and increase caregiver burden, often leading to institutionalization (9, 10). The ability to successfully address these neuropsychiatric symptoms has the potential to prolong independent living at home, delay institutionalization and may reduce caregiver burden, and reduce expenses.

## Pharmacological approaches to neuropsychiatric symptoms of AD

The Food and Drug Administration (FDA) had not approved pharmacological interventions for treatment of AD neuropsychiatric symptoms until the recent approval of brexipiprazole (Rexulti) (11). The vast majority of pharmacological treatments for AD neuropsychiatric symptoms are prescribed off-label. Most commonly, physicians prescribe atypical antipsychotic medications to address psychosis in AD, but these have black-box warnings, potentially making them unsafe (12, 13). Although brexipiprazole was approved for the treatment of agitation associated with AD, it carries a black-box warning for increased mortality in elderly individuals with dementia-related psychosis as well as increased risk of suicidal thoughts and behaviors in young adults (11). Additionally, off-label prescribed medications often have side effects that may be difficult to tolerate in elderly populations with comorbidities such as cerebrovascular conditions and Parkinson’s disease. Common psychotropic medications including olanzapine, quetiapine, risperidone are typically discontinued within 3 months because of lack of tolerability or associated cerebrovascular events and extrapyramidal symptoms (14). Moreover, because of the large spectrum of neuropsychiatric symptoms in AD, treatments to address depression, agitation, psychosis, or sleep disturbances often lead to polypharmacological prescribing and pose an increased risk for side effects. Thus, non-pharmacological treatments, including music-based interventions, can constitute first-line psychosocial treatments that may maintain neuropsychiatric regulation without the risk of side effects (2, 15).

## Music to address AD neuropsychiatric symptoms

Researchers have provided a neurological rationale for music as a psychosocial intervention for AD. For example, Ferreri et al. conducted a double masked within-subject design in healthy participants engaged in music listening and showed a positive association between dopamine and the music experience. The participants received a dopamine precursor (levodopa), a dopamine antagonist (risperidone), or a placebo (lactose) (16). The authors found that the dopamine precursor levodopa enhanced the hedonic and motivational experience associated with music, whereas the dopamine antagonist diminished the effect. In a separate study, Wang et al. evaluated fMRI resting-state data from the Alzheimer’s Disease Neuroimaging Initiative (ADNI) to study functional connectivity within and between auditory and rewards systems in older adults with mild cognitive impairment (MCI), AD, and age-matched healthy controls (17). Wang et al. found preserved within- and between-network connectivity in the auditory and reward systems in MCI individuals compared to AD, suggesting that functional connectivity is impaired as AD pathology progresses.

Scholars conducting preliminary studies in music and AD have indicated that music ability and memory are housed in an “island of preservation” in people with dementia (18). Memory for familiar music can be somewhat maintained in AD (19–23). The ability to respond, recall, or produce music by singing, playing instruments, or composing is often preserved even in the severe stages of AD (24, 25). When 7 Tesla functional brain magnetic resonance imaging (fMRI) and positron emission tomography (PET) compared AD regions of interest to the brain’s response to music excerpts, regions such as the anterior cingulate cortex and ventral pre-supplementary motor area which encoded musical memory corresponded to areas, showed substantially minimal cortical atrophy and minimal disruption of glucose-metabolism (26). The unique relationship between music and preserved memory suggests music-based interventions, including music therapy (MT), may constitute an approach to address neuropsychiatric symptoms in AD.

## Introduction to music therapy

MT is a specific music-based intervention wherein qualified music therapists (MT-BCs^1^) use music to address clinical objectives within the context of a therapeutic relationship. MT is based on the interactions between the service user,^2^ a compassionate and empathetic MT-BC, and the music. Capitalizing on the associations between music, neuroscience, and the interpersonal relationship between the service user and therapist, MT can be an engaging psychosocial treatment for people with AD. In the United States, MT-BCs undergo academic training and accrue 1,200 supervised clinical training hours prior to the board-certification exam. Resulting from their “rigorous” (27) academic and clinical training, MT-BCs are uniquely qualified to design and implement effective psychosocial interventions for people with AD to address their diverse needs. MT-BCs are educated in and guided by psychotherapeutic frameworks that are developmentally and clinically appropriate given the service user, objectives, and context. MT-BCs are knowledgeable and skilled in a plethora of music genres and styles, various instruments and voice, and the psychology of music (28, 29). With this unique knowledge and skill set, MT-BCs implement research-based interventions to maximize therapeutic outcomes.

After receiving a referral from a healthcare professional, the MT-BC conducts a formal assessment as part of the treatment process to design and tailor interventions that best use the person’s strengths and motivations to address their needs. MT interventions are based on the service user’s assessment, music preferences, experiences, and motivations for therapy and therefore minimize the potential for music-induced harm (30). Tailored MT interventions can vary depending on factors related to the service user’s preferences and experiences, the MT-BC, and music all within the unique contextual parameters of the setting and related clinical objectives. As such, MT interventions typically vary across different service user populations, clinical objectives, as well as the education, clinical experiences, and approaches of the MT-BC.

Regarding the music within MT, there are often misperceptions that certain music genres are beneficial as well as that there are music genres more likely to result in detrimental impacts on people’s health (31–36). However, each individual’s preferred music is the most effective and therapeutic regardless of the genre or message within the music (37, 38). Resultant of its malleability and the MT-BC’s musical skill sets, live music can be more effective and therapeutic than recorded music (39, 40). Therefore, MT-BCs are academically and clinically trained to be knowledgeable in a wide variety of music genres and are competent musicians on instruments including voice, piano, guitar, and percussion. The use of preferred live music during interactive MT can also result in a stronger therapeutic relationship, alliance, and therapeutic outcome (30, 41).

MT is distinct from receptive music listening and music medicine^3^ as MT-BCs address non-musical clinical objectives that have been collaboratively formulated by the service user, MT-BC, and the multidisciplinary treatment team. MT-BCs integrate service user’s preferred music and present it in a developmentally appropriate manner. Moreover, MT-BCs use live music with optimized levels of repetition to enhance engagement and clinical success and are able to manipulate a variety of musical elements including melody, harmony, tempo, dynamics, timbre, and structure. Common MT interventions for people with AD may include singing, playing instruments, composition, reminiscing, and receptive music listening. MT-BCs are trained to use nonverbal behavior with older adults and those with AD to enhance clinical outcomes (42, 43). The gestalt of these music and common therapy factors based on traditional talk-based interventions can result in augmented service user engagement, motivation, and positive treatment outcomes (38, 42, 44, 45).

Further supporting MT for AD, music is processed in bilateral cerebral hemispheres and is considered to be different from noise. Stegemöller et al. (2018) proposed that professionally trained musicians such as MT-BCs have less noise in their speech and singing (46). As a result, Stegemöller et al. (2018) suggested that MT can augment neuroplasticity because the brain is more efficient at processing a clear auditory signal (46). Additionally, the Neuroplasticity Model of Music Therapy (NMMT) describes how MT can augment neuroplasticity via utilizing the service user’s preferred music (46, 47). The NMMT and Hebbian principle note that pairing novel information and behaviors with rhythm can synchronize neural activation and augment the likelihood of neuroplasticity, particularly seen when MT-BCs are able to successfully manipulate various elements of live music by activating various brain regions (46). As such, music and MT may be an effective way to engage people with AD and thus offer potential therapeutic effects for the neuropsychiatric symptoms associated with AD.

To date, the existing MT literature for AD is positive but limited in its scope related to neuropsychiatric symptoms. Moreover, there is a paucity of literature regarding music teletherapy (MTT) research outcomes. This gap in the literature is consequential because MTT may be able to increase access to MT as a psychosocial intervention to address neuropsychiatric symptoms in AD. Therefore, the purpose of this paper is to provide a rationale for a protocolized music teletherapy (MTT) intervention to address neuropsychiatric symptoms in AD by conducting a narrative review of the existing MT and AD literature.

## Method

### Narrative review

To provide a rationale for a protocolized MTT intervention to increase accessibility for MT as a psychosocial intervention for neuropsychiatric symptoms in people with AD, we conducted a focused search via PubMed and Google Scholar. Inclusion criteria consisted of refereed AD articles published in English using the neuropsychiatric inventory (NPI) (48) as a dependent variable. The NPI is a frequently used quantitative measurement for neuropsychiatric symptoms available in over 40 different languages and has been used in 350 clinical trials. The NPI examines many of the neuropsychiatric changes that develop in AD including delusions, hallucinations, agitation/aggression, depression, anxiety, elation/euphoria, apathy/indifference, disinhibition, irritability, aberrant motor behavior, sleep and night time behavior disorders, appetite and eating disorders (48–50). Tailoring our review to the NPI allowed us to compare the studies in a more standardized manner. We included AD but excluded other forms of dementia. We recognize these criteria as delimitations of the paper.

### Results and rationale for music teletherapy

MT is considered beneficial and safe by people with AD and their caregivers (51). We identified eight existing clinical trials that met our inclusion criteria of MT services to treat neuropsychiatric symptoms of individuals with AD. We extracted relevant data from these studies and depicted the results in Table 1. As seen in Table 1, most researchers investigating MT found significant improvement on neuropsychiatric symptoms of AD. However, there are limitations in these studies. The researchers conducting these trials did not use a single standardized MT protocol and the approaches towards how MT was conducted varied widely.

**Table 1:** MT clinical trials for treatment of neuropsychiatric symptoms in AD.

Author(s)	Design	Sample	Independent variable	Music therapy	Dependent measure(s)	Results
Brotons and Marti, 2003 (52)	Within subjects.	*N =* 14 couples (Patient and spouse who is a caregiver). Probable diagnoses of AD, Stages 4–5 GDS.	Individuals with AD participated in 10 MT sessions, individuals with AD and caregivers participated in 7 sessions together, and caregivers alone in 4 sessions.	MT for individuals with AD involved in music listening, singing, instrument playing, and movement/dance. MT sessions of individuals and caregivers included instrumental ensembles and singing. Caregivers alone engaged in singing, music listening, music relaxation, musical games, and song writing. Two professional music therapists, including one board-certified music therapist (MT-BC).	NPI and other measures taken at baseline, 2 days before the end of MT, and 2-months post.	Three time points indicated a lower NPI score (X2 = 17.72, *p* = 0.001).
Gallego et al. (2021) (53)	Quasi-experimental. 6 nursing homes were masked and randomized to 1 of 3 conditions.	*N =* 90. Active music involved groups of 6, 7, 8, and 9 residents. Receptive music involved groups of 6, 7, and 8 people. Control had groups of 8, 9, 11, and 12 residents.	45-min. group tx twice a week for three months (12 sessions total). Active music vs. receptive music vs. control group who watched nature videos without any music.	Tx consisted of active music intervention, receptive music intervention, or usual care. Active music and receptive music contained the same songs except the opening and goodbye songs. Active music included rhythmic exercises, dance exercises, music games. Receptive music listening involved listening to a playlist from a computer with a facilitator naming the song title, performer’s name, and providing residents the opportunity to reminisce. Music facilitators, with master’s level-qualification in creative arts therapy and specialization in MT.	NPI and other measures taken at baseline and post.	NPI decreased in the active music (*p* = 0.001), did not change in receptive music, and increased in control group (*p* = 0.001).
Gómez Gallego and Gómez García 2017 (54)	Within subjects.	*N* = 42. 25 mild and 17 moderate AD.	MT group tx twice a week for 45-min. across 6 weeks.	Welcome song, rhythmic accompaniment with clapping and instruments, movement to music, musical games, and goodbye song. Included two professionals trained in music therapy.	NPI and other measures taken at baseline, 6th session (3 weeks), and final session.	Decrease in NPI total scores for both mild and moderate AD.
Giovagnoli et al. (2018) (55)	Randomized controlled trial.	*N =* 45. 23 exp. and 22 control.	MT group twice a week for 40 min. across 24 weeks.	Active music therapy by a music therapist. Non-verbal approach and improvisational	NPI and other measures taken at baseline, 12, and 24 weeks.	Decrease in NPI at week 12 for experimental group, *p* = 0.039. Between group differences in NPI was *p* = 0.253. Less patients in AMT showed worsening of NPI score at 24 weeks compared to control group, *p* = 0.048.
Li et al. (2015) (56)	Quasi-experimental trial design. Separated into MT or control groups according to acceptance of adjunct MT or not.	*N =* 41. 20 in MT and 21 in control.	Individualized music listening at home for 30 min. Daily in the morning and before sleep at night across 6 months.	Receptive, listening-based MT. Consisted of excerpts of Mozart’s Sonata for Two Pianos in D major (KV 448) in the morning for 30 min. and Pachelbel’s Canon in D major for violins at night. Did not mention a music therapist involved in the study.	NPI and other measures taken at baseline and 6 months (as tx ended).	No significant difference between the two groups; MT had less behavioral and psychological symptoms than control group after cognitive status was adjusted.
Lyu et al. 2018 (57)	Randomized controlled trial.	*N* = 288 completed. 96 mild, 100 moderate, and 95 severe AD. 97 in group singing, 96 in lyric reading, and 95 in control group.	Singing group, lyric reading of favorite or familiar song without music, or control. 2x per day for 30 to 40-min. per session, for 3 months.	Singing or listening to favorite & familiar songs. Did not mention a music therapist involved in the study.	NPI and other measures taken at baseline, 3 months (as tx ended), and 6-months post tx.	Singing group had greatest reductions in NPI, significant difference. While moderate had even better NPI scores, those with severe in group singing had the greatest improvement across time, both at the end of the tx and 6-months post tx.
Raglio et al. 2008 (58)	Non-standardized randomization criteria. Participants listed in alphabetical order and those who were listed as odd numbers = experimental group. Even = control group.	*N* = 59. 30 exp. and 29 control.	30 30-min. group MT sessions across 16 weeks vs. educational support or entertainment activities.	Use of rhythmic and melodic instruments to promote communication. Briefly mentions a music therapist involved in the study but does not define any qualifications or training designations.	NPI and other measures taken at baseline, 8, 16 (end of tx), and 20 weeks (4 weeks post tx).	NPI scores decreased in experimental group but not control group (interaction x group: *F*_3, 165_ = 5.06, *p* = 0.002. Differences between the 2 groups occurred at 8th (*p* = 0.003), 16th (*p* < 0.0001), and 20th weeks *p* = 0.0007).
Satoh et al. (2015) (59)	Recruited 10 to experimental group. Then recruited 10 more who were willing to participate but could not be due to inclusion or exclusion criteria.	*N* = 20. 10 exp. and 10 control. Mild to moderate AD.	1 h, MT group session once a week for 6 months vs. control.	Singing training utilizing voice training (YUBA Method), reviewing songs from previous week, singing familiar songs with normal voicing, and singing familiar songs from youth or recent years as part of a life review. Karaoke was used for all but the voice training. Also required to practice 20 min. at home with a karaoke system 3 x per week at home. Authors were a professional singer and pianist who led the MT sessions.	NPI and other tests along with fMRI baseline and posttest.	Decrease in NPI score, *p* = 0.042 for music therapy group.

AD, Alzheimer’s disease, exp., experimental, min., minutes, tx, treatment, GDS, Geriatric Depression Scale.

### Lack of standardized music therapy protocols

Although MT can be beneficial as well as safe for people with AD and their caregivers, current MT practice is heterogeneous with variable therapist qualifications and therapeutic approaches (51, 60–62). For example, although there are 99 MT training programs in Europe, the European MT confederation reports that standardization of training standards have not yet been completed (63). MT interventions can also vary between recorded music versus live music. Additionally, there can be differences in active interventions including instrument playing, composition, music making, singing, listening, and reminiscing. The totality of the vast number of musical elements to consider, MT intervention types, and sociocultural aspects of music further compound the heterogeneity of MT. Moreover, MT researchers have not consistently protocolized intervention approaches, making it difficult to objectively assess the state of the literature.

The protocolization of MT may help to standardize it as a nonpharmacological AD treatment option, improve dissemination, and incorporate it into standard of care for AD. To develop effective protocols that will lead to referrals and increase access to care, it will be crucial to develop systematic and reproducible measures to identify mechanisms of change including but not limited to dosage, duration, procedures, and MT intervention components that predict clinically significant improvement in AD neuropsychiatric outcomes. To date, researchers have not empirically identified therapeutic process factors that contribute to MT outcomes in AD. Thus, it is imperative to identify critical MT process elements to enable future treatment refinement and to train MT-BCs to reproduce high-quality MT, both of which ultimately may improve patient outcomes. In addition, researchers will need to report these mechanisms and components in a transparent manner such that standardized reproducible protocols are developed and accepted as standard of care. As such, we recommend using reporting guidelines for music-based interventions (64) and clearly articulating the qualifications, approach, and experiences of the practitioner providing MT. Protocolized music teletherapy (MTT) intervention has the potential to overcome some of the barriers associated with MT and increase accessibility such that MT becomes a realistic and viable psychosocial intervention option for neuropsychiatric symptoms in people with AD.

### Music therapy access

There are approximately 10,000 MT-BCs in the United States. However, as MT is a medium-specific profession and MT-BCs serve a variety of clinical populations, not all MT-BCs work in AD settings. Within the United States, the AD population is currently at 6.5 million and predicted to reach over 12.5 million by 2050 (65, 66). Given these statistics, it is unlikely that there will be enough MT-BCs to meet the psychosocial needs of people with AD.

In addition to a limited number of MT-BCs and a growing population of people with AD, 50% of people with AD stop driving within 3 years of disease onset (67). Thus, challenges in transportation logistics in AD can further limit MT access. The amalgamation of these factors severely restricts the ability of MT-BCs to provide in-person MT.

We therefore recommend MTT as an option to increase access to services by eliminating the need for patients to drive to sessions. With MT-BCs providing care remotely, it would also eliminate time allocated to driving. The reduction in driving may lead to the ability to provide additional services. By eliminating travel times, MT-BCs may also increase their billable hours and increase their earning potential. Increased revenue may lead to fewer MT-BCs leaving the profession (68–70).

### Music therapy awareness

To date, there is limited research regarding MT for neuropsychiatric symptoms related to AD. This lack of research based on standardized protocols likely impacts the awareness of MT as a potential treatment for AD. Moreover, care providers are often not aware of MT as a psychosocial intervention for AD because of poor access to MT in the outpatient setting, and therefore do not make referrals (71). Additionally, it is possible caregivers are unaware of MT as a treatment for AD and therefore are not likely to request it as a treatment option for their loved ones. Reasons that caregivers are unaware of MT as a treatment in the USA may be because the United States’ Alzheimer’s Association website does not offer MT as a treatment option (72).Whilst the National Institute of Aging recommends music and singing to patients with AD, the NIA does not specifically recommend MT as a treatment for neuropsychiatric symptoms in AD (73). As a result of the combination of these factors, patients and caregivers can experience difficulty in obtaining MT services.

### Rationale for protocolized music tele-therapy

To date, there is no published clinical trial study investigating MTT for neuropsychiatric symptoms in AD. However, authors have noted that non-MT telehealth interventions can be delivered for people with MCI and AD and that these treatments can be as effective as in-person delivery (74–77). MT scholars reported that MT can be delivered to older adults via telehealth (78, 79). Although MTT was already in existence (80, 81), it was popularized out of necessity during the COVID-19 pandemic (82). Various MT authors have described MTT as a potential service delivery model (78, 79). Telehealth can reduce caregiver burden, reduce access barriers, and reach a wide range of patients in rural areas or locations with low numbers of music therapists (83). Furthermore, MTT augments accessibility and reduces travel time for both the therapist and service users. Music therapists are likely to continue using telehealth in the future and believe that caregiver involvement is important (78).

Given the interactive and music-related aspects of MT, there are potential complications with MTT including but not limited to compromised quality of the music, delays when interacting or when concurrently engaging in live music, reliable internet connections, and secure and accessible platforms. These complications may be exacerbated in AD populations who may have difficulty learning new skills such as accessing MTT on a phone, tablet, or computer. However, advances in technology have made it easier for individuals to use smartphones and tablets for therapeutic purposes (84, 85). We suggest providing service users with high quality instruments, technology that relies on cellular data instead of home-based wifi, and using both live and recorded music and more talk-based therapy approaches in MTT. As approximately 50% of surveyed music therapists reported that they would continue telehealth delivery after the pandemic restrictions are over (79), MTT is a viable delivery format for MT.

During the pandemic, researchers stated there was a need to determine how people with AD and their caregivers benefit from MT services delivered via telehealth and the role of the caregiver in the process (78). Currently, there is no music therapy study comparing an in-person delivery format with MMT; however, other non-MT telehealth psychosocial interventions for older adults with dementia and their caregivers can be as effective as in-person delivery (75–77). Based on these results, MTT may have potential to be as effective as in-person service delivery formats. Relatedly, Saragih et al. suggested future researchers conduct trials to determine what factors are associated with positive outcomes in telehealth interventions for people with dementia and their caregivers as there is a need to provide evidence for what might be effective with older adults with dementia as well as the mechanisms of action. Based on our review, we suggest this is also the case for MT and MTT.

**Figure 1:**
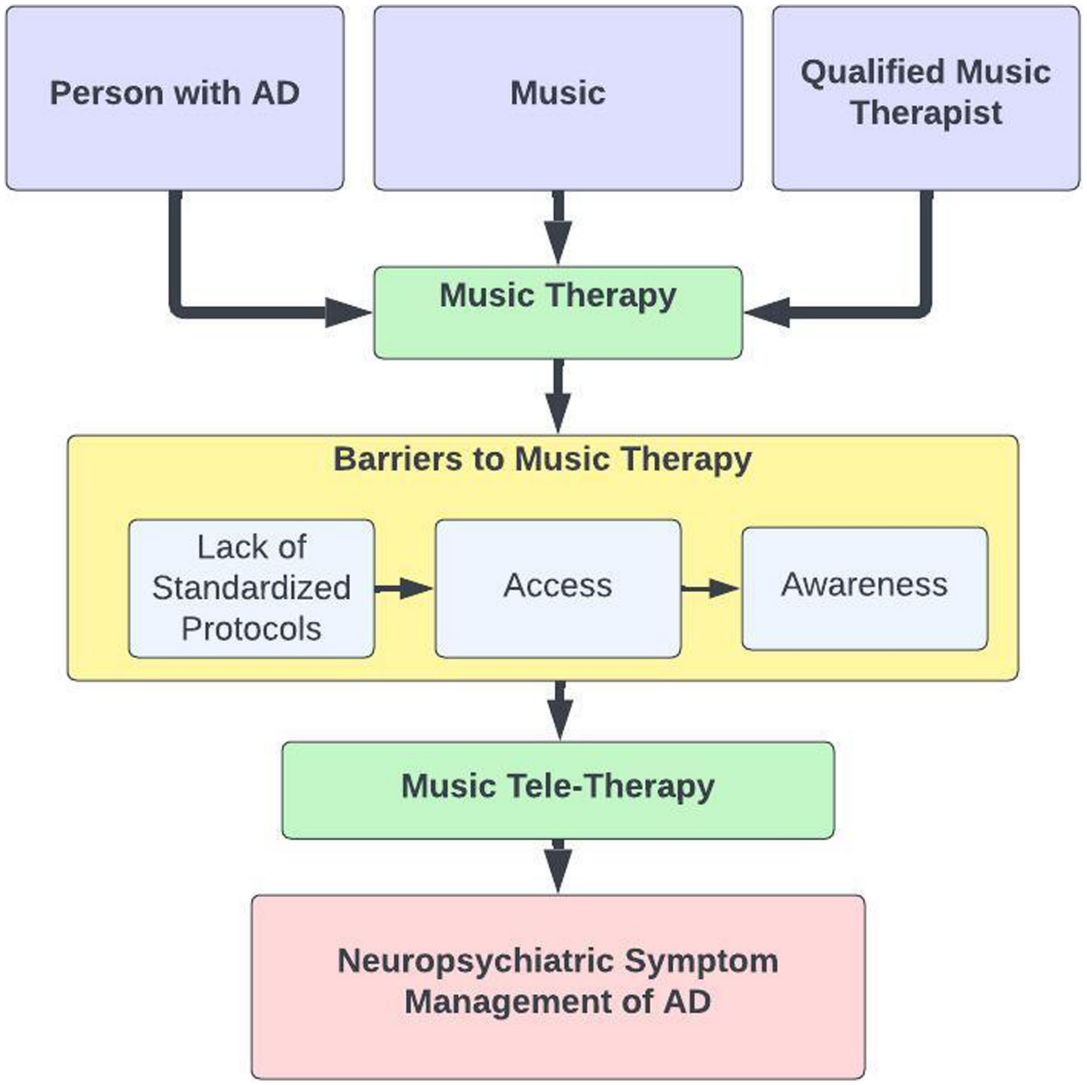
Barriers to MT for AD utilization and delivery. A flow diagram depicts the barriers to music therapy and music tele-therapy as a solution in the management of neuropsychiatric symptoms of AD.

Our narrative review regarding MTT to increase accessibility for MT as a psychosocial intervention for neuropsychiatric symptoms in AD implicates three main categories representing barriers to MT for AD utilization and delivery: (1) lack of standardization in MT protocols, (2) lack of access to music therapy, and (3) lack of awareness. Based on the interactions between these identified factors, we created Figure 1 to depict a rationale for MTT to address neuropsychiatric symptoms in AD.

The barriers to accessing music therapy are problematic, especially since music might be a viable non-pharmacological approach to address the neuropsychiatric symptoms for adults with AD. We therefore propose music therapists utilize MTT to increase the number of people served, particularly those with AD and their caregivers, who often have limitations in accessing treatment. As a person-centered and flexible treatment, both MT and MTT have the potential to address the neuropsychiatric symptoms that are problematic for people with AD and their caregivers. However, MTT has the potential to improve access to care.

## Limitations

There are numerous limitations of this review. First, our inclusion criteria were purposely narrow as we wanted to specifically investigate the impact of MT on neuropsychiatric symptoms as measured by the NPI in AD. There were other articles wherein authors investigated music and MT for individuals with dementia or addressed different dependent measures (86–90). Although using the NPI as inclusion criteria allowed us to compare and contrast studies with a validated quantitative outcome measure, it also limited our results to eight studies. Only including articles published in English is also a limitation and we note the privilege associated with our familiarity of the English language. A final and consequential limitation is that not all countries have established MT training programs or qualified MT practitioners.

### Suggestions for future research

Based on the narrative review, there is a need for studies to improve the understanding of the underlying mechanisms of MT via imaging and biomarkers. To date, the underlying mechanisms of action within MT for AD are poorly understood and a better comprehension of these mechanisms may lead to the ability to design best practice MT interventions that may include neuromodulation or pharmaceuticals to augment MT’s clinical effects. MT may have the potential to raise people’s thresholds for tolerating environmental stimuli that activate unmet needs in AD (91). Therefore, future investigators could design and measure the impact of MT interventions and structured MT environments to address unmet needs by adjusting the sensory input and maximizing skills and abilities of each individual throughout the session (44). Future research using broader inclusion criteria may identify limitations and help develop a research agenda. Additionally, researchers could study process elements within MT to identify what components of MT are most clinically significant such that these processes are incorporated into standardized protocols. These may include specific features related to the music, the therapist, as well as specific music therapy interventions. Future researchers might also examine the differences between music medicine and receptive music listening and MT provided by a MT-BC. This is a crucial item on the research agenda to protect service users from music induced harm (30). However, given the challenges that people with AD may have, we recommend MT/MTT because of the specialized academic and clinical training that MT-BCs receive. Therefore, it would be beneficial for researchers and clinicians to compare MTT to MT as well as other established treatments by measuring neuropsychiatric symptoms. Future service delivery model research is also warranted to compare MT with MTT. For example, the most user-friendly approaches to MTT application should be studied to optimize the clinical impact of music therapy. These suggestions for future research may also consist of clinical trials, effectiveness, feasibility, mechanistic, and refinement studies.

## Conclusion

The purpose of this paper was to provide a rationale for a protocolized MTT intervention to increase accessibility for MT as a psychosocial intervention for neuropsychiatric symptoms in people with AD. We conducted a narrative review of MT publications using the NPI as a dependent measure indexed in PubMed and Google Scholar. Based on the narrative review of eight studies that met our inclusion criteria, MT seems to have positive impacts on neuropsychiatric symptoms in AD. However, we identified an ensuing need for protocolized MT interventions, increased access to MT, and greater awareness of MT. As a relatively inexpensive psychosocial intervention, MTT can be an accessible option with the potential to address these barriers. Although MT can have beneficial effects on neuropsychiatric symptoms in AD, we highlight a subsequent need for MT that is easily accessible and follows a standardized intervention protocol. MTT has the potential to constitute a viable solution to fulfill these needs. Future MTT research from all paradigms is necessary.

## Author contributions

SW, AC-T, MS, and SY conceived the concept of the review, performed the research and wrote the manuscript. All authors contributed to the article and approved the submitted version.

## Footnotes

^1^We use MT-BC to represent qualified practitioners, as we are geographically located in the United States, and this is the official qualification for music therapists (www.cbmt.org). We recognize that other countries have different terminologies for professional qualification.

^2^We recognize that people with AD can be referred to as patients, clients, or service users depending upon a number of contextual factors including but not limited to setting, country, level of care, and relationship to care provider.

^3^Typically occurring in adult medical settings, music medicine can be defined as listening to recorded music that is chosen by a medical professional without specialized music training, assessments, or a therapeutic process (Dileo, 1999; Silverman et al., 2016; Yinger & Gooding, 2014).
